# Bacterial outer membrane vesicles provide an alternative pathway for trafficking of *Escherichia coli* O157 type III secreted effectors to epithelial cells

**DOI:** 10.1128/msphere.00520-23

**Published:** 2023-11-06

**Authors:** Natalie Sirisaengtaksin, Eloise J. O'Donoghue, Sara Jabbari, Andrew J. Roe, Anne Marie Krachler

**Affiliations:** 1 Department of Microbiology and Molecular Genetics, McGovern Medical School, The University of Texas Health Science Center at Houston, Houston, Texas, USA; 2 School of Biosciences, Institute of Microbiology and Infection, University of Birmingham, Edgbaston, Birmingham, United Kingdom; 3 School of Mathematics, Institute of Microbiology and Infection, University of Birmingham, Edgbaston, Birmingham, United Kingdom; 4 School of Infection and Immunity, College of Medical, Veterinary and Life Sciences, University of Glasgow, Glasgow, United Kingdom; The University of Iowa, Iowa City, Iowa, USA

**Keywords:** outer membrane vesicles, EHEC, OMV, vesicular trafficking, secretion systems

## Abstract

Outer membrane vesicles (OMVs) are proteoliposomes shed by Gram-negative bacteria. Their secretion is enhanced by the transition into the intra-host milieu, and OMVs play critical roles during pathogenesis. Enterohemorrhagic *Escherichia coli* O157 (EHEC) can cause diarrheal disease in humans, and soluble toxins including Shiga-like toxins that contribute to disease severity and clinical complications like hemolytic uremic syndrome have been shown to be OMV associated. In addition to Shiga-like toxins, EHEC produces a type III secretion system (T3SS), and T3SS effectors are associated with colonization and disease severity *in vivo*. Here, we show that type III secreted substrates including translocators and effectors are incorporated into OMVs both in the presence and absence of a functional T3SS. EHEC strains with non-functional T3SS shed more OMVs, and vesicles enter host cells with accelerated kinetics compared to vesicles shed from wild-type EHEC. The T3SS effector translocated intimin receptor (Tir) is trafficked from OMVs into host cells and localizes to the membrane. However, its clustering on the host membrane and co-localization with bacterial pedestals is intimin dependent. We show that OMV-delivered Tir can cross-complement an effector-deficient EHEC strain and promote pedestal formation. Together, these data demonstrate that OMVs provide an alternative pathway for the delivery of EHEC T3SS cargo and that vesicle associated effectors are released from OMVs inside host cells and can retain biological activity.

**IMPORTANCE:**

Bacteria can package protein cargo into nanosized membrane blebs that are shed from the bacterial membrane and released into the environment. Here, we report that a type of pathogenic bacteria called enterohemorrhagic *Escherichia coli* O157 (EHEC) uses their membrane blebs (outer membrane vesicles) to package components of their type 3 secretion system and send them into host cells, where they can manipulate host signaling pathways including those involved in infection response, such as immunity. Usually, EHEC use a needle-like apparatus to inject these components into host cells, but packaging them into membrane blebs that get taken up by host cells is another way of delivery that can bypass the need for a functioning injection system.

## INTRODUCTION

Enterohemorrhagic *Escherichia coli* (EHEC) are a leading cause of food-borne diarrheal disease worldwide. In some cases, gastrointestinal symptoms can be complicated by the development of hemolytic uremic syndrome (HUS), which is associated with increased morbidity and mortality (1). Secreted Shiga-like toxins lead to the development of HUS, and the accessory toxins cytolethal distending toxin V (CdtV) and hemolysin (Hly) are thought to contribute to HUS pathology (2, 3). However, many more virulence factors are associated with primary colonization of the gastrointestinal tract, in particular the locus of enterocyte effacement (LEE), which encodes for a type III secretion system (T3SS) and associated effectors (4, 5). The T3SS is a needle-like conduit that translocates effector proteins into the host cell where they manipulate host cellular signaling, induce cytoskeletal rearrangements, and modulate immunity to facilitate infection. The T3SS needle is formed by the structural protein EspA (6), and secretion activity is driven by the ATPase EscN (7). EscN directly interacts with T3SS chaperones as well as secreted effectors (8). The T3SS initially translocates structural proteins, followed by the translocon components EspD and EspB which perforate the host cell membrane (9, 10), and in response to environmental signals, eventually switches to secrete effectors targeting host proteins (11, 12).

The translocon components EspB and EspD form a complex (13) that embeds into and forms a pore in the host membrane bilayer (14, 15). EspB is both part of the translocon as well as an effector. It has been shown to also be targeted to the host cell cytoplasm (10), where it interacts with catenin and myosin to cause actin reorganization and contribute to microvillus effacement and inhibition of phagocytosis (16, 17). Other T3SS effectors include the translocated intimin receptor (Tir), which inserts into the host membrane and contains both intracellular and extracellular domains. The protein is clustered in the membrane via interactions with the adhesin intimin (18), and Tir clustering leads to downstream signaling events that ultimately result in actin reorganization and pedestal formation (18, 19). Tir is critical for colonization and disease pathogenesis in the infant rabbit model (20).

In addition to type III secretion systems, which are pathogen specific, Gram-negative bacteria also use a range of generalized secretion systems to transport cargo. Outer membrane vesicles (OMVs) are thought to be an alternative secretion system that can facilitate both inter-bacterial (21, 22) as well as bacteria-to-host cargo transfer (23). OMVs are proteoliposomes of 10 to 200nm diameter formed by budding of the outer membrane and can contain membrane-associated and soluble proteins, nucleic acids, and small molecules. OMV production is a constitutive process but is also a protective response and increases in the presence of environmental stress and during infection (24–27). Often, this increase in OMV production is co-regulated with other virulence mechanisms (24, 28).

EHEC-derived OMVs play important roles during infection: they constitute a decoy protecting bacteria against antimicrobial peptides (24) and have been shown to enter host cells (29). EHEC OMVs traffic a wide range of cargoes into host cells, including lipopolysaccharide (30, 31), Shiga-like toxins, CdtV, and Hly (29, 32), which have been shown to reach the target cells in a biologically active form and contribute to pathogenesis (29, 31). However, localization and trafficking of T3SS components by OMVs have not been studied. Here, we set out to investigate whether T3SS components could be trafficked by OMVs and whether this process may act as an alternative pathway to facilitate effector delivery to host cells.

## RESULTS

### Type III secreted proteins localize to outer membrane vesicles in the presence and absence of type III secretion activity

**Fig 1:**
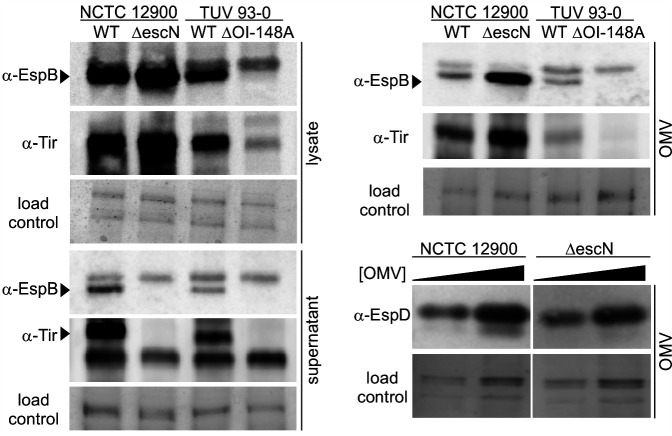
Type III secreted proteins localize to outer membrane vesicles in the presence and absence of functional type III secretion. Total cell lysates, supernatants, and OMVs were harvested from NCTC 12900 wt and an isogenic ∆*escN* deletion strain or a TUV 93-0 wt and an isogenic ∆OI-148A deletion strain. All fractions were normalized using OD_600_ readings at harvest. For EspD blots, OMV concentrations were measured and adjusted to concentrations of 10^11^ (left lanes) and 2 * 10^11^ OMVs/mL (right lanes) for both strains. All fractions were separated by SDS-PAGE and either probed by Western blotting using α-EspB, α-Tir, or α-EspD antibodies to visualize the translocon components EspB and EspD and the effector Tir or stained with Coomassie blue to provide loading controls.

First, we set out to determine whether EHEC OMVs contain proteins that are usually secreted through the type III secretion machinery. Type III secretion is a hierarchical process, where needle components are secreted as early substrates, followed by secretion of the translocon protein EspD and EspB and finally the effectors. We measured the amount of the translocator EspB as well as the T3SS effector Tir in total cell lysates, culture supernatants, and purified OMVs from EHEC wild-type strain NCTC 12900 and the isogenic secretion-deficient mutant ∆*escN*, which lacks the translocation ATPase. We also probed fractions of EHEC wild-type strain TUV 93-0 and the isogenic mutant ∆OI-148A. ∆OI-148A contains a deletion of the first half of the LEE pathogenicity island, which removes the important regulator Ler required for expression of the T3SS (33). As previously shown, all strains except the ∆OI-148A mutant produced EspB and Tir (Fig. 1). As expected, secretion was only detected in both wild-type strains, but not in the ∆*escN* and ∆OI-148A mutants. Purified OMVs contained both translocon components EspB and EspD, as well as the effector Tir, and localization of the T3SS components to OMVs required the presence of the T3SS locus but did not depend on a functional T3SS, since OMVs collected from the ∆*escN* mutant also contained EspB, EspD, and Tir (Fig. 1).

### Loss of type III secretion activity leads to hypervesiculation

**Fig 2:**
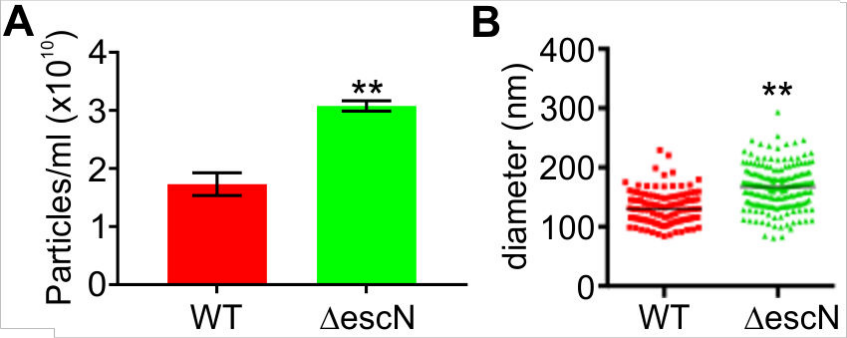
A type III secretion deficient EHEC strain produces excess outer membrane vesicles. OMVs were isolated from an equal biomass of NCTC 12900 wt (red) or ∆*escN* (green) cultures grown in LB for 18 hours at 37°C. OMV concentrations (**A**) and OMV size distributions (**B**) were measured using a Nanosight LM10 particle tracking system. A minimum of 100 tracks per sample were determined for three individual preparations per strain. Statistical significance was determined using unpaired *t*-tests and is depicted with asterisks. Symbol ** indicates a *P*-value ≤0.01.

To determine the effect of T3SS activity on OMV production, EHEC wild-type and a T3SS secretion-deficient strain, ∆*escN*, were compared. The ∆*escN* strain produces T3SS structural components and effectors but is unable to secrete them, since the T3SS ATPase EscN is lacking (34). Both strains were grown in lysogeny broth (LB) for 18 hours, culture densities were normalized, and OMVs were harvested and quantified using Nanosight particle tracking analysis. Both wild-type and ∆*escN*-derived OMVs were free from bacteria and low-molecular-weight debris. The ∆*escN* mutant produced significantly more OMVs per cell than the NCTC 12900 wild-type strain (Fig. 2A). The mean diameter of vesicles produced by the isogenic ∆*escN* strain was slightly larger than that of OMVs derived from the corresponding wild-type strain (Fig. 2B).

### Outer membrane vesicle uptake by host cells is accelerated in the absence of a functional type III secretion system

**Fig 3:**
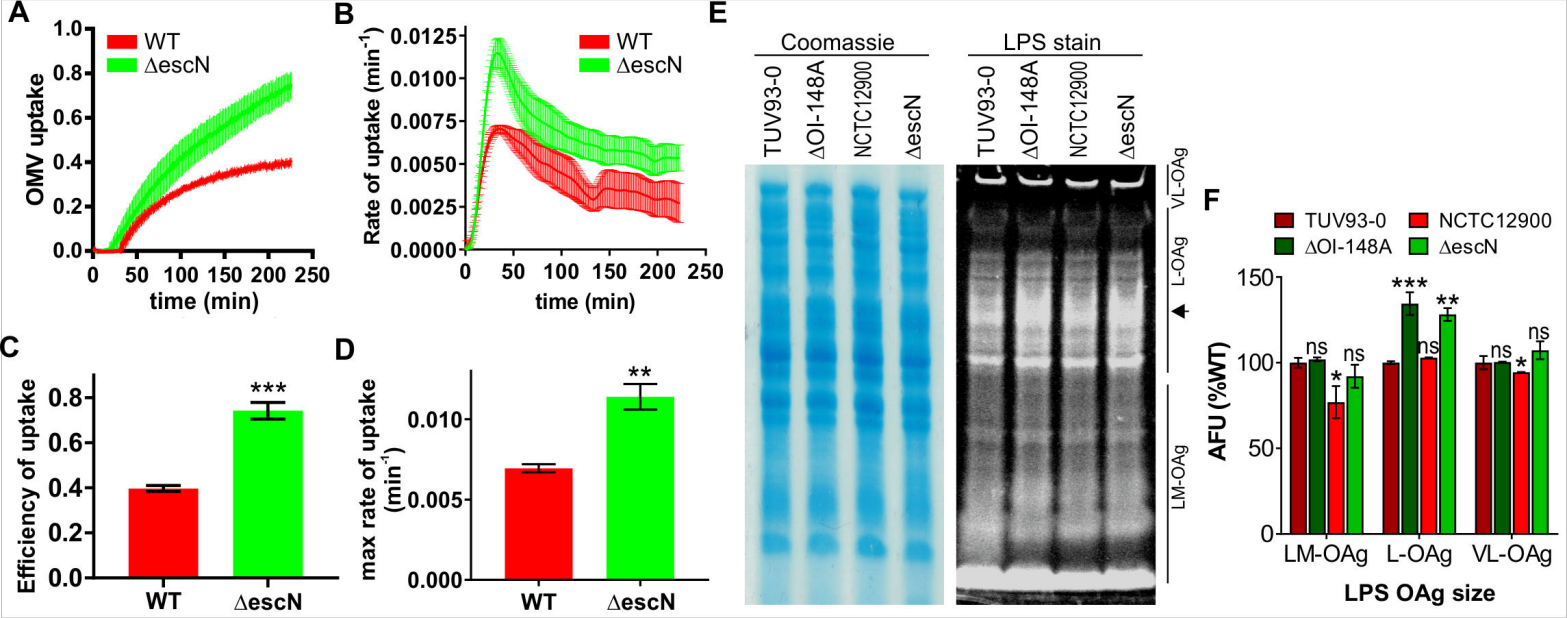
Outer membrane vesicle uptake by host cells is accelerated in the absence of a functional type III secretion system. (**A**) CCF2-AM loaded HeLa epithelial cells were exposed to OMVs from EHEC NCTC 12900 wt (red) or a ∆*escN* deletion strain (green) carrying ClyA-Bla at an MOI of 1,000 for 3.5 hours. Ratios of blue:green fluorescence over time were plotted as means ± SEM (*n* = 3). Rates (**B**) were extracted from data in panel A to visualize speed of OMV uptake over time and are means ± SEM (*n* = 3). FRET signal changes after 3.5 hours (uptake efficiency) (**C**) and maximum rates of uptake (**D**) were determined from data in panels **A** and **B**, respectively. Data shown are means ± SEM (*n* = 3). Significance was determined by unpaired *t*-test, with **P ≤ 0.01 and ***P ≤ 0.001. Cultures of EHEC TUV 93-0 wt or T3SS-deficient mutant ∆OI-148A, NCTC 12900 wt, or T3SS secretion-deficient mutant ∆*escN* were separated by SDS-PAGE, and total protein and LPS were visualized by Coomassie brilliant blue and Pro-Q Emerald 300 staining, respectively. VL-OAg, very long O-antigen; L-OAg, long O-antigen; LM-OAg, low and medium length O-antigen. Arrow on right marks region of difference between wild-type and mutant strains. (**F**) Quantification of LPS content by densitometry of stained gels. Arbitrary fluorescence units are normalized using Coomassie-stained gels, and intensities for each LPS population are expressed relative to the TUV93-0 wild-type strain (100%). Data are means ± SEM (*n* = 3). Significance was determined using one-way analysis of variance (ANOVA) and Brown-Forsythe test; ns, not significant; **P ≤ 0.01 and ***P ≤ 0.001.

To determine if loss of a functional T3SS would affect the kinetics of OMV entry into host cells, we used a CCF2-AM-based reporter assay as previously described (30). This assay uses a vesicle-targeted beta-lactamase reporter (ClyA-Bla) to measure the fast entry kinetics of OMVs into the host cell cytoplasm in real time. ClyA is a cytolysin produced by pathogenic *E. coli*, is naturally incorporated into OMVs, and therefore acts as a targeting component for the enzymatically active TEM domain of β-lactamase (Bla). Epithelial host cells were pre-loaded with the fluorescent beta-lactamase substrate CCF2-AM, which upon enzymatic cleavage shifts from green to blue fluorescence emission (30, 35). The ClyA-Bla reporter was expressed in either EHEC NCTC 12900 wild-type cells or the isogenic ∆*escN* mutant. OMVs were harvested, and the concentration of both OMV samples was standardized to a multiplicity of infection (MOI) of 1,000 OMVs per cell, using concentration data from particle tracking analysis. As previously determined (30), this corresponds to an MOI of approximately 37 bacteria per cell, a physiologically relevant level. OMVs were incubated with dye-loaded epithelial cells, and uptake kinetics were measured for three and a half hours (Fig. 3A and B). Both wild-type and ∆*escN-* derived OMVs were rapidly taken up by host cells, but OMVs from ∆*escN* were taken up with higher efficiency (Fig. 3C) and significantly faster (Fig. 3D) than vesicles derived from the wild-type strain. We conclude that in the absence of a functional T3SS, OMV uptake is accelerated. Since we have previously established that the chemical structure of the bacterial lipopolysaccharide plays a significant role in rate and efficiency of OMV uptake (30), we analyzed the LPS structure of NCTC 12900 wild-type and ∆*escN* strains. As a control, we also analyzed the LPS of the EHEC strain TUV 93-0 and a corresponding isogenic mutant (∆OI-148A) that does not express the T3SS (33). While the short and very long O-antigen chains were similar in all four backgrounds, both the ∆*escN* and ∆OI-148A featured slightly longer O-antigen chains than either wild-type strain (Fig. 3E, arrow, and Fig. 3F). Together, these data suggest that in the absence of a functional T3SS, the lipopolysaccharide composition is altered. Based on the previously observed link between LPS composition and entry pathways (30), these differences in LPS between wild-type and T3SS mutant could account for the differences in uptake kinetics observed here. Additionally, the absence of a functional T3SS could potentially cause changes in the composition of OMV cargo or localization of cargo within OMVs that lead to altered host receptor binding and, consequently, OMV uptake dynamics. Further studies to explore this are needed.

### Outer membrane vesicles get delivered to host cells independent of type III secretion activity

**Fig 4:**
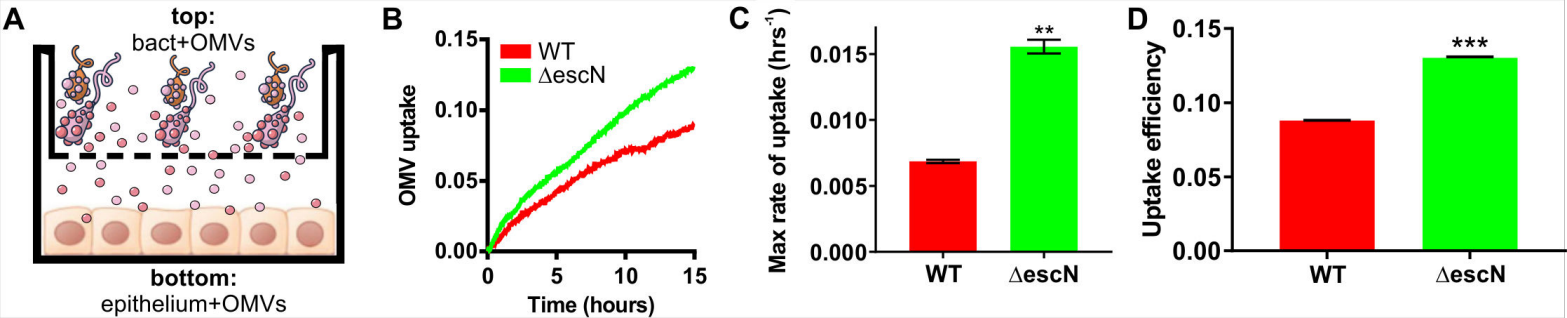
Outer membrane vesicles get delivered to host cells *in vivo* independent of type III secretion activity. (**A**) Experimental setup for OMV release and delivery experiments. Epithelial cells are grown in the bottom compartment of a transwell and loaded with CCF2-AM dye. Bacteria (MOI = 10) are added to the top compartment of the transwell, and OMV uptake by host cells in the bottom well is monitored by measuring FRET over time. (**B**) CCF2-AM loaded HeLa cells were exposed to EHEC NCTC 12900 wt (red) or a ∆*escN* deletion strain (green) carrying ClyA-Bla, at an MOI of 10 for 15 hours. Ratios of blue:green fluorescence over time were plotted as means ± SD (*n* = 3). Maximum rates (**C**) were extracted from data in Fig. 4B to visualize speed of OMV uptake and are means ± SD (*n* = 3). FRET signal changes after 15 hours (**D**) were determined from data in panel** B** and plotted to visualize overall efficiency of uptake. Data shown are means ± SD (*n* = 3). Significance was determined by unpaired *t*-test, with **P ≤ 0.01 and ***P ≤ 0.001.

Although the use of purified OMVs for infection experiments allowed us to normalize the number of vesicles used, the system is somewhat artificial, in that it does not follow the physiological time course of vesicle formation, release, and diffusion to host cells. To test if OMVs could be taken up by host cells without prior purification and enrichment, we set up a transwell experiment, where bacteria were grown in the transwell top compartment to allow OMV production and release, and host cells were cultured in the bottom compartment to permit contact with OMVs following diffusion through the transwell but exclude access and direct contact with intact bacterial cells (Fig. 4A). Uptake of OMVs by host cells was then measured using CCF2-AM loaded epithelial cells and ClyA-Bla expressing EHEC strains. OMV uptake was observed over 15 hours, since OMV uptake was significantly slower than those observed with isolated OMVs (Fig. 4B). We observed a decrease in uptake efficiency (Fig. 4D) compared to purified, concentrated OMVs, likely because OMV formation and release as well as vesicle concentration are additional time-limiting factors. Nevertheless, maximum rates of uptake (Fig. 4C) were similar than those of purified concentrated OMVs (Fig. 3D). Overall, these data suggest that OMV cargo is delivered to host cells via OMVs, despite a lack of direct bacteria-to-host cell contact.

### The T3SS effector Tir is delivered to host cells via OMVs

**Fig 5:**
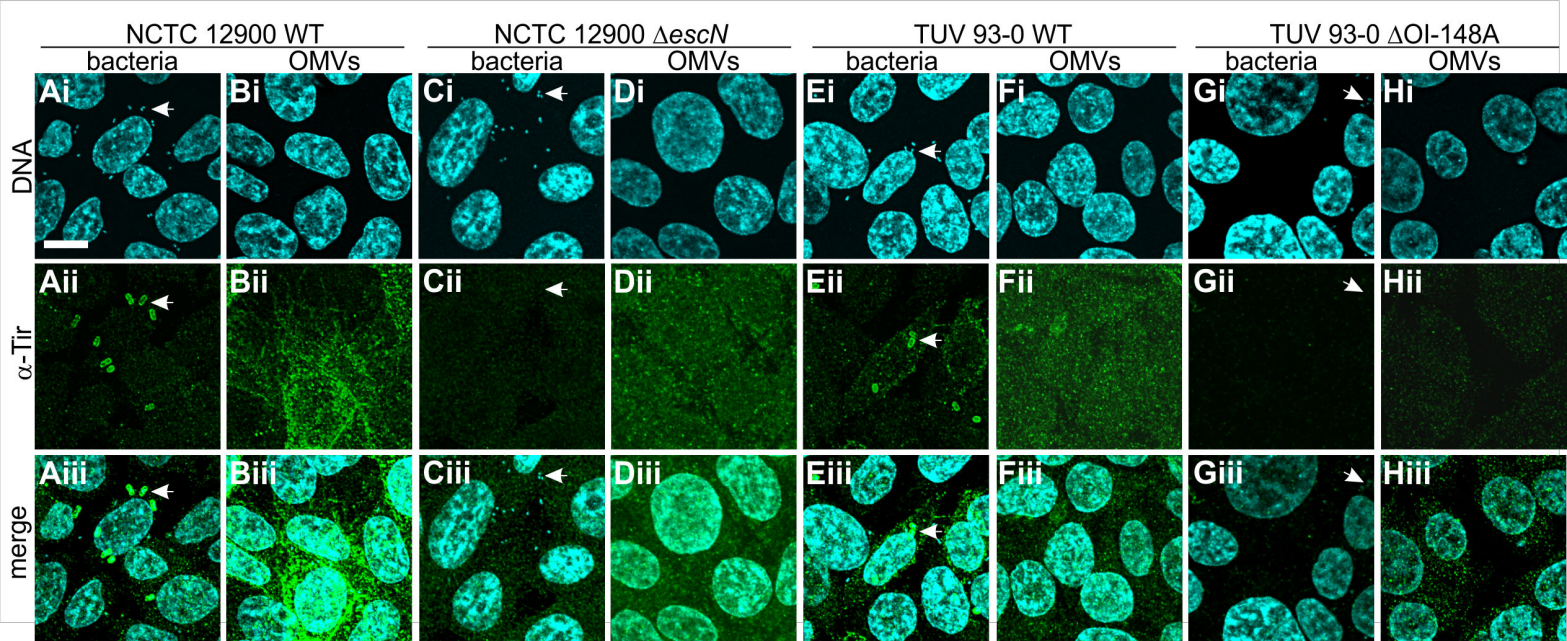
The T3SS effector Tir is delivered to host cells via OMVs. Intestinal epithelial (RKO) cells were infected with bacteria at an MOI of 30 (**A, C, E, G**) or purified OMVs at an MOI of 1,000 (**B, D, F, H**) for 4 hours. Strains used were NCTC 12900 wild-type (**A and B**), NCTC 12900 ∆*escN* (**C and D**), TUV 93-O wild-type (**E and F**), and TUV 93-O ∆OI-148A (**G and H**). RKO cells were fixed in paraformaldehyde (PFA), permeabilized with Triton X-100, and stained. Images of Hoechst to visualize DNA (i), α-Tir antibody to visualize translocated intimin receptor (ii), and merged images (iii) are shown. Images are representative of *n* = 3 experiments. Scale bar, 10 µm. Arrows mark examples of individual bacteria.

Since we established that T3SS effectors associate with OMVs, we set out to test if OMV-associated effectors would be trafficked into host cells. Intestinal epithelial (RKO) cells (ATCC CRL-2577) were infected with wild-type or secretion-deficient (∆*escN*) bacteria or wild-type and T3SS-deficient (∆OI-148A) derived OMVs for 4 hours, at an MOI of 37 bacteria per cell or a corresponding MOI of 1,000 OMVs/cell. Host cells were then fixed and stained with α-Tir antibody and Hoechst to visualize translocated intimin receptor within host cells and DNA, respectively (Fig. 5).

In intestinal epithelial cells infected with wild-type bacteria (NCTC 12900, Fig. 5A, or TUV 93-0, Fig. 5E), Tir was found throughout the host cell and was particularly concentrated in foci (pedestals) below attached bacteria. In contrast, Tir was absent from cells infected with bacteria deficient in T3SS secretion (∆*escN*, Fig. 5C) or deficient in T3SS expression (∆OI-148A, Fig. 5G). When cells were infected with OMVs derived from both wild-type strains as well as the secretion-deficient mutant ∆*escN*, Tir was present in host cells. However, its localization was not focal but diffuse all over the plasma membrane (Fig. 5B, D, and F). Tir was absent following incubation with OMVs derived from the ∆OI-148A strain which does not express the T3SS (Fig. 5H). We also tried to stain cells using an α-EspB antibody, but the signal was too weak to draw conclusions, even after prolonged incubation. Together, our data suggest that effectors usually secreted via the EHEC T3SS can also be delivered to intestinal cells via outer membrane vesicles.

### OMV-delivered translocated intimin receptor reaches the host cell in a biologically active form

**Fig 6:**
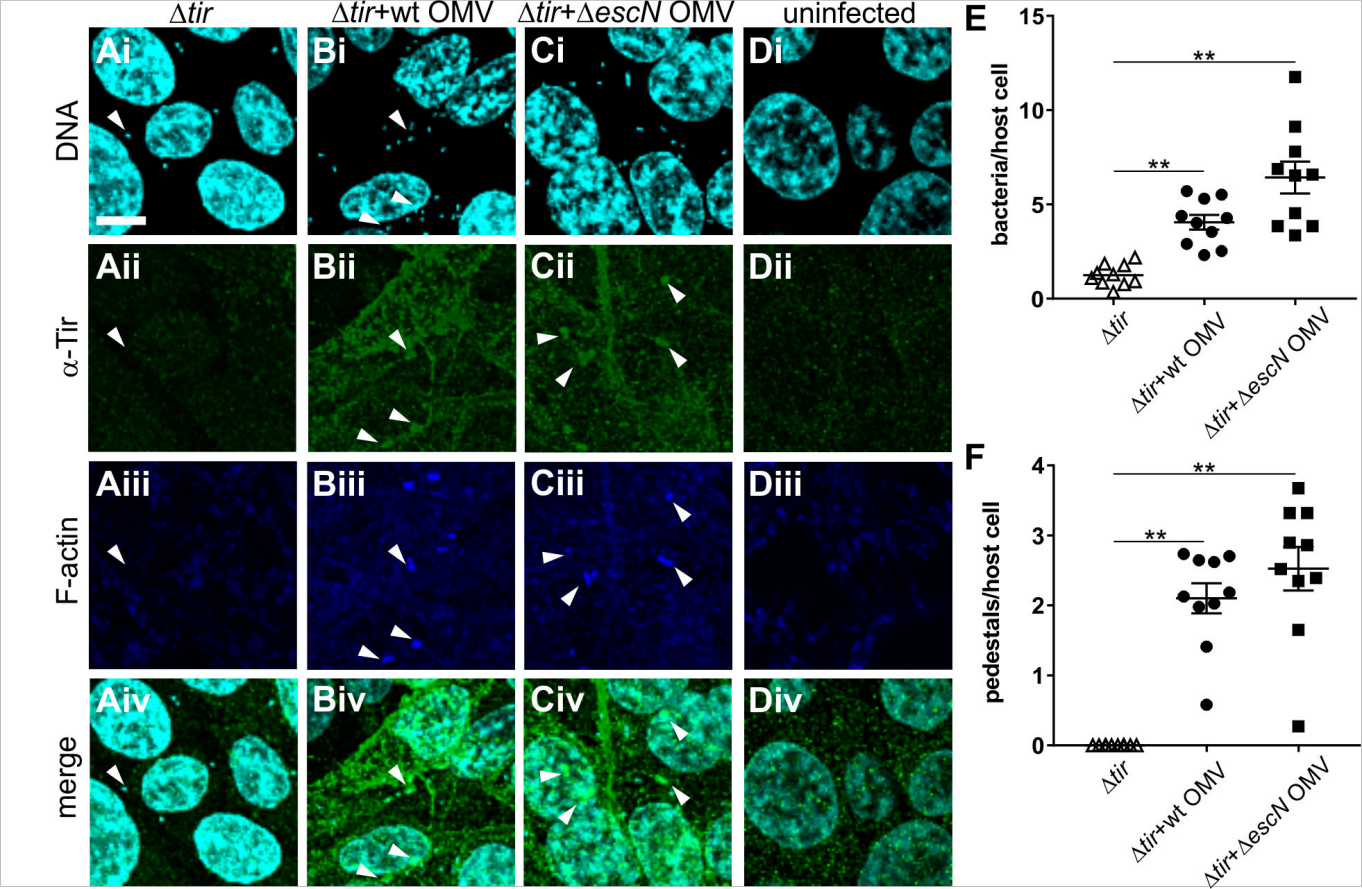
OMV delivered Tir can restore pedestal formation by Tir-deficient EHEC. Intestinal epithelial (RKO) cells were infected for 4 hours with bacteria (EHEC ∆tir) at an MOI of 30 either alone (**A**) or in the presence of OMVs at an MOI of 1,000 derived from NCTC 12900 wild-type (**B**) or ∆*escN* mutant (**C**). As a control, RKO cells were left uninfected (**D**). RKO cells were fixed in PFA, permeabilized with Triton X-100, and stained. MIP images of Hoechst to visualize DNA (i), α-Tir antibody to visualize translocated intimin receptor (ii), phalloidin to stain F-actin (iii), and merged images (iv) are shown. Images are representative of *n* = 3 experiments. Scale bar, 10 µm. Arrows mark examples of individual bacteria. (**E**) Bacteria per host cell and (**F**) pedestals per host cell were quantified from individual z-slices. Individual counts, means, and SEM from 10 slides (a total of ≥500 RKO cells) across three independent experiments are shown. Significance was analyzed using one-way ANOVA and Brown-Forsythe test. Symbol ** indicates a corrected *P*-value ≤0.01.

Since we observed that the T3SS effector Tir could be trafficked into intestinal cells via OMVs, we next asked whether Tir would be released from the OMVs and retain its biological activity. We infected intestinal epithelial cells with a Tir-deficient EHEC strain, which attaches to host cells but fails to form pedestals (Fig. 6A). We then asked whether Tir-containing OMVs could cross-complement and reconstitute pedestal formation for the Tir-deficient strain. Tir was absent from cells infected with EHEC *∆tir* (Fig. 6A) but present when infections were carried out in the presence of OMVs derived from wild-type or ∆*escN* strains (Fig. 6B and C). The presence of bacteria caused the redistribution of OMV-derived Tir, which formed foci under the bacterial cells (Fig. 6B and C). No Tir or pedestals were observed for uninfected cells (Fig. 6D). Overall, bacterial attachment was enhanced, and actin pedestal formation, restored in the presence of OMV-derived Tir (Fig. 6E and F). Since insertion of Tir into the host cell membrane is a prerequisite for the formation of actin foci, these data suggest that the T3SS effector Tir is released from the vesicles and associates with the host membrane in its biologically active conformation.

## DISCUSSION

Outer membrane vesicles are abundantly produced by Gram-negative bacteria, and OMV production has been shown to enhance the pathogenic potential of bacteria, including EHEC O157. OMVs isolated from EHEC O157 have been shown to contain a range of virulence factors, including Shiga-like toxins and hemolysin, which are traditionally thought of as secreted toxins (36, 37). EHEC OMVs are sufficient to cause hemolytic uremic syndrome-like symptoms (38) and are a key activator of pro-inflammatory responses to bacterial infections *in vivo* (31). OMVs additionally play important roles during infection, including as a decoy against host-secreted antimicrobial peptides (24). OMVs are constantly produced, but the transition to the intra-host milieu and exposure to cues such as colonic medium and mucin significantly increases vesiculation. OMV production is highest in host-isolated EHEC and decreases during lab cultivation (39). Similarly, antibiotic treatment increases OMV production and the amount of OMV-associated Shiga-like toxins (39). These quantitative differences between OMV production in laboratory culture and *in vivo* are likely the reason why OMV-mediated trafficking of T3SS effectors has thus far eluded detection in conventional *in vitro* culture assays.

In addition to Shiga-toxins, type III secreted effector proteins are critical factors promoting EHEC colonization and pathogenesis *in vivo* (4). T3SS effectors modulate key cellular signaling processes, leading to cytoskeletal rearrangements and modulation of host immune responses. In this study, we set out to investigate whether type III secreted proteins are also secreted via outer membrane vesicles and whether they would be biologically active within the host.

OMV biogenesis is thought to require local instabilities in the bacterial cell envelope—either the local loss of linkage between outer membrane and peptidoglycan layer or local changes in the outer membrane organization, such as changes in phospholipid content or curvature (40). Here, we found that failure to assemble a functional T3SS also leads to increased vesiculation (Fig. 2). The ATPase-deficient ∆*escN* mutant assembles the type III secretion system basal body but is incapable of producing a needle and secreting effectors. The T3SS structure traverses both membranes as well as the peptidoglycan layer, and T3SS assembly is known to be accompanied by local remodeling of the peptidoglycan layer (41, 42). It is conceivable that the initiation of peptidoglycan remodeling by T3SS-associated lytic transglycosylases without concurrent full assembly of the T3SS leads to local changes in envelope structure that result in increased vesiculation. The mechanistic details of this process have yet to be elucidated in future studies.

We report here that EHEC OMVs contain T3SS associated factors (Fig. 1), including the translocon components EspB and EspD, as well as the effector Tir—factors which are ordinarily moved from the bacterial cytoplasm to the host cell. Although current models of OMV biogenesis depict vesiculation as a process of outer membrane blebbing, it is well known that OMVs contain cytoplasmic factors as well as periplasmic and outer membrane components. EHEC-derived OMVs have been shown to contain bacterial DNA, including genomic DNA encoding for virulence factors (36). Although the finding that EHEC T3SS-associated substrates are contained in OMVs is novel, Salmonella T3SS components have been shown to be secreted via OMVs (43, 44). Additionally, substrates for other secretion systems have previously been found associated with OMVs. For example, *Pseudomonas aeruginosa* incorporates type VI secreted substrates into OMVs to facilitate iron acquisition (45). OMVs of the Gram-negative oral pathogen *Tannerella forsythia* are enriched with substrates of the type IX secretion system (46). Interestingly, Ernst et al. recently identified a chaperone-independent secretion pathway for T3SS effectors in the intestinal pathogen *Shigella*, although the involved pathway was not subjected to detailed analysis (47). It would be interesting to see whether the identified pathway is consistent with OMV-dependent effector secretion.

We further found that the uptake of EHEC OMVs derived from a secretion-deficient *∆escN* T3SS mutant was accelerated compared to OMVs from the wild-type strain (Fig. 3). We have previously described that the preferred uptake pathway and thus entry kinetics of OMVs are shaped by a strain’s lipopolysaccharide composition (30). Although we identified minor changes in the long O-antigen of T3SS-deficient strains compared to wild-type isolates (Fig. 3) which would be consistent with the accelerated uptake kinetics, it is unclear whether those changes are sufficient to explain the accelerated entry kinetics we observed here. It has been described previously that LPS composition and T3SS activity can impact each other (48, 49) and that O-antigen length is inversely correlated with the amount of T3SS expression (50). Additionally, it is possible that the T3SS needle structure facilitates targeting to the clathrin-mediated endocytic route, which mediates slower trafficking to the cytoplasm, and that the absence of the needle structure from both mutants drives preferential uptake via raft-mediated endocytosis, which enables faster and more efficient trafficking of OMV contained substrates to the cytoplasm, as we have described previously (30).

We observed that OMV-associated T3SS effectors reach the host cell cytoplasm and retain their endogenous biological activity in the context of an infection (Fig. 5 and 6). The delivery of biologically active virulence factors we observe here is in agreement with other studies that found OMV-delivered Shiga toxin, hemolysin, and cytolethal distending toxin V all retain biological activity within host cells, despite being subject to endosomal trafficking (29). We found that OMV-delivered Tir preferentially localizes to the host cell membrane, both when translocated through the T3SS and when delivered via OMVs (Fig. 5). However, the characteristic focal pattern we observe upon whole cell infection (Fig. 5A and E) is absent when the effector is delivered via OMVs, where we instead observe diffuse localization on the host plasma membrane (Fig. 5B, D and F). However, co-localization with bacteria and with actin pedestals is restored for vesicle-trafficked Tir, once bacteria attach to the host cell (Fig. 6). This indicates that the effector remains biologically active, but its correct localization is dependent on the presence of attached bacteria and is most likely induced by the presence of its cognate ligand, intimin. This idea is consistent with previous work on EPEC Tir and intimin, where the formation of ring-like supramolecular structures was induced in Tir-primed host cells by the addition of intimin containing proteoliposomes (51). We also observed that Tir delivered to host cells appears to be enriched around cell-cell contacts (Fig. 5). EHEC OMVs have recently been described to transiently disrupt tight junctions and translocate through intestinal cells via both paracellular and transcellular pathways. This has been attributed to the overall pro-inflammatory effect of OMVs rather than any one particular factor included in the OMVs (52).

Together, our data provide evidence for the trafficking of biologically active type III secreted effectors to epithelial cells by outer membrane vesicles, an alternative pathway for the delivery of EHEC effectors to host cells. It is conceivable that this pathway is relevant during infection *in vivo*, where the amount of bacterial vesiculation is significantly higher than observed under laboratory conditions. It has already been demonstrated by several groups that EHEC OMVs significantly contribute to pathogenicity *in vivo* (31, 38). To what extent the OMV-mediated delivery of T3SS substrates to host tissues is important for virulence *in vivo* will be the subject of future studies.

## MATERIALS AND METHODS

### Bacterial strains and growth conditions

The strains used in this study were the *E. coli* serotype O157:H7 strain NCTC 12900, an STX-negative isolate (NCTC12900), and the isogenic T3SS ATPase-deficient ∆*escN* mutant (53, 54), as well as strain TUV 93-0 and isogenic deletions ∆OI-148A and ∆*tir* (33). For studying entry kinetics, bacterial strains were transformed with plasmid pBAD-ClyA-Bla (kan^R^), a kanamycin-resistant derivative of the pBAD amp^R^ vector (55) provided by Matthew DeLisa, Cornell University. Strains were grown in LB containing 50 µg/mL kanamycin for selection where necessary, at 37°C with shaking at 200 rpm. ClyA-Bla expression was induced by adding 0.02% arabinose to the growth medium.

### Isolation of outer membrane vesicles

The detailed procedure for OMV isolation has been published before (30). Briefly, for isolation of OMVs, strains were grown in LB for 18 hours. Bacterial cells were pelleted by centrifugation at 6,000 × *g*, and supernatants, filtered through 0.45-µm filters. Filtrate was plated to ensure sterility. OMVs were pelleted from 25 mL of filtrate by ultracentrifugation at 100,000 × *g* for 2 hours at 4°C, washed with DMEM, and resuspended in 1 mL of sterile DMEM until further use. Alternatively, OMVs were purified from culture supernatants using Exo-spin columns (Cell Guidance Systems) following the manufacturer’s protocol.

### Nanosight analysis of outer membrane vesicles

To measure concentrations and analyze sample purity via scattering, purified vesicles were diluted 1:10^6^ in filtered phosphate buffered saline (PBS), and OMV concentrations and diameters were determined using a Nanosight LM10 particle tracking system. A minimum of 100 tracks were determined per sample, with analysis performed on three separate preparations per sample. Original concentrations were calculated and normalized to cell densities. Prior to infection experiments, OMV preparations were diluted in DMEM to normalize them to an MOI of 1,000 (corresponding to an MOI of 37 bacteria per cell).

### Western blotting

EHEC strains were grown in LB for 18 hours at 37°C. Cells were harvested by centrifugation and normalized to an OD of 10 in 5× SDS-PAGE loading buffer and boiled. Ten microliters were analyzed by SDS-PAGE to determine contents of total cell lysates by Western blotting. Supernatants were concentrated by acetone precipitation, and amounts normalized to cell densities were analyzed by Western blotting. OMV fractions were concentrated and normalized to give a concentration of 10^10^ particles/mL, and 10 µL was analyzed by SDS-PAGE. Gels were transferred to a PVDF membrane, blocked for 1 hour at 22°C in Tris-buffered saline (TBS) 5% skimmed milk. Membranes were probed with primary antibodies (α-EspD 1:2,000), α-Tir (LS-C500576 from LSBio, 1:1,000), or α -EspB (ABIN 1098174, 1:1,000) in blocking buffer at 4°C for 16 hours and secondary α-mouse HRP (1:5,000) for 1 hour at 22°C. Bound antibodies were detected using Pierce ECL Plus Western Blotting Substrate.

### Infection experiments

HeLa cells (ATCC CCL-2) were seeded at 10^5^ cells/mL into black-walled, clear-bottom 96-well plates 24 hours prior to infections. On the day of the experiment, cells were loaded with CCF2AM for 1 hour at 22°C as per the manufacturer’s instructions and then washed, and media were replaced with supplement-free DMEM. Cells were incubated with OMVs from EHEC to give an MOI of 1,000 (corresponding to a cell-based MOI of approximately 37), and blue and green fluorescence were monitored using a PheraStar Omega plate reader, using excitation at 405 nm and simultaneous dual emission at 530 nm and 460 nm, every 2 minutes as previously described (30).

For transwell infection experiments, HeLa cells were seeded in 24-well plates in complete DMEM at 10^5^ cells/mL 24 hours prior to infections. Cultures of EHEC NCTC 12900 and ∆*escN* were grown in LB and induced with 0.02% L-arabinose. Two hundred microliters of cultures were added to the transwell inserts, with 600 µL of DMEM without supplements added to the cells in the bottom compartment. Plates were analyzed using a PheraStar Omega plate reader for 15 hours, monitoring blue and green fluorescence with excitation at 405 nm and simultaneous dual emission at 530 nm and 460 nm. All experiments were performed with a minimum of three technical replicates and three independent repeats. The ratio of blue to green fluorescence intensity detected in the cells at each cycle was calculated for each experiment.

### Rate estimation, efficiency of uptake, and statistical analysis

To estimate the gradients of the data (i.e., rates of uptake), polynomials were fitted to each data set using the cubic spline function *csaps* in Matlab R2017b. Numerical estimates of the gradients of the resulting polynomials were determined using the *gradient* function. To ensure that the gradient estimates were as smooth as possible while also retaining the overall shape and trend of the data, a small smoothing parameter was used. Analysis of variance (ANOVA) was used to determine statistical significance, with a Brown-Forsythe test to determine equal variance (GraphPad Prism software). A *P*-value of <0.05 was considered statistically significant. Efficiency of uptake was calculated as the absolute change in blue:green fluorescence intensity ratio between 0 and 3 hours ([Em460/Em530]_t=0h_)/[Em460/Em530]_t=3h_). ANOVA was used to determine statistical significance, with a Brown-Forsythe test to determine equal variance (GraphPad Prism software). A *P*-value of <0.05 was considered statistically significant.

### Immunofluorescence staining and microscopy

Infections were carried out as described above, with the exception that RKO cells (ATCC CRL-2577) were used and seeded onto glass cover slips. Cells were infected with EHEC or OMVs as described above for 4 hours, washed with PBS, and fixed with 3.2% paraformaldehyde in PBS at 4°C for 16 hours. Fixed cells were permeabilized with 1% Triton X-100 in PBS for 10 minutes and blocked using 1% bovine serum albumine (BSA) in TBST for 1 hour. Cells were then stained with α-Tir (LS-C500576 from LSBio, 1:1,000 in blocking buffer) for 1 hour at room temperature, washed three times with TBST, and stained with α-rabbit secondary antibody conjugated to Alexa-488 (1:500 in blocking buffer) for 1 hour at room temperature. Cells were washed three times with TBST, stained with Hoechst (1:1,000 in PBS), and phalloidin conjugated with Alexa-680 (1:100 in PBS) for 10 minutes and washed three times with PBS and one with water prior to mounting in ProLong Antifade Gold. Slides were imaged after curing in the dark for 16 hours, using an Olympus IX83 with a Fluoview FW3000 confocal system and a 100× oil immersion objective.
